# The use of visual methods to explore how children construct and assign meaning to the “self” within two urban communities in the Western Cape, South Africa

**DOI:** 10.3402/qhw.v11.31251

**Published:** 2016-06-09

**Authors:** Elizabeth Benninger, Shazly Savahl

**Affiliations:** Department of Psychology, University of the Western Cape, Bellville, South Africa

**Keywords:** Self, self-concept, child well-being, Photovoice, community maps, visual methods

## Abstract

This study aimed to explore how children construct and assign meaning to the “self” within two urban communities of Cape Town in South Africa. Using a child participation methodological framework data were collected using Photovoice and community maps with 54 participants between the ages of 9 and 12. Feelings of safety, social connectedness, and children's spaces were found to be central to the ways in which the participants constructed and assigned meaning to the “self.” The study provides implications for intervention programmes aimed at improving children's well-being to be inclusive of activities aimed at improving children's self-concept, including the construction of safe spaces for children to play, learn, and form meaningful relationships.

The ways in which children construct and assign meaning to the “self” plays a central role in a young person's psychosocial functioning and their overall well-being. In Social Psychology, the way children think about their traits, abilities, and attributes is commonly referred to as the self-concept (Egan & Perry, 1998). More broadly, it is perceived as a multi-dimensional construct created as a reflection of a child's interaction with their social environment (Kenny & McEachern, 2009), which is largely shaped by the institutions, culture, history, and the social conditions in which they live (Staub, 2003). For example, conditions of poverty and oppression could limit the resources useful to a young person to nurture the development of a healthy self-concept. This is especially apparent within violent areas, where a child's underlying sense of safety is compromised (Savahl, Isaacs, Adams, Carels, & September, 2013). Such conditions exist within the townships of the Western Cape in South Africa, where the repercussions of Apartheid's system of structural racism has resulted in the continuity of impoverished and violent living conditions.

Although the history of systematic violence in South Africa has its genesis in the colonization era in the 1600s, violence in the form of structural racism became normalized during the Apartheid regime (1948–1994), which segregated the South African society into four distinct racial groups (“Black,” “Coloured,” “Indian,” and “White”) premised on “White” privilege and superiority (Lockhat & Van Niekerk, 2000). During this time, the residents classified as “Black” and “Coloured” were forcibly removed to townships located on the periphery of the city. These townships were poorly resourced in terms of infrastructure, services, and access to education and healthcare. The violence from the oppressive conditions led to a normalization of violence within the communities, where neighbourhoods, schools, and homes turned into spaces of conflict, rather than nurturing environments for the local children (Lockhat & Van Niekerk, 2000). The oppressive and violent conditions created during Apartheid continue to have an impact upon the well-being of the local children with potentially damaging repercussions on their self-concept. Recent statistics reveals 50% of South African children live in conditions of poverty and violence (UNICEF, 2009; World Bank, 2013), which compromises their well-being, sense of self, and opportunities to play safely (Savahl et al., 2013).

## Children's well-being and self-concept

Well-being is defined as a holistic outlook best understood through an integration of both objective and subjective components such as life satisfaction, income, nutrition, and the way people think and feel about issues related to their health and happiness (White, 2008). The inclusion of both the objective and subjective components of well-being contributes to a different and deeper understanding of the construct (Axford, Jodrell, & Hobbs, 2014), which must be located within a particular context and centred in the person and his or her own perspectives and priorities (White, 2008).

Prior research has defined well-being as an umbrella term to encompass specific concepts and indicators, such as a positive self-concept (Camfield, Streuli, & Woodhead, 2009). Further research has identified the self-concept to have a strong influence on children's subjective well-being (see Camfield, 2012; Fattore, Mason, & Watson, 2007; McLean, Pasupathi & Pals, 2007; Schimmack & Diener, 2002). For example, the child participants from Savahl et al. (2015) noted a *stable self* to be critical to a positive self-identity and their overall well-being. Schimmack and Diener (2002) compared the predictive validity of explicit and implicit self-esteem measures for informant reports of subjective well-being and found explicit self-esteem to be a predictor of subjective well-being. Based within the township of Ocean View, South Africa, Moses (2006) highlighted the complexity of the interaction of neighbourhood features with each other and with the broader physical, economic, and socio-political context and its impact on children's well-being in terms of access to resources, hampering integration, and impact on children's self and collective-efficacy.

The above research points to the importance which the self-concept plays in our understanding of children's well-being. Key to this process is the understanding of how children make sense of and assign meaning to the self. The current study hopes to contribute in this regard by providing an exploration of how children construct and assign meaning to the self within two urban communities of the Western Cape through the use of various methods that capture the views and perspectives of the children themselves. This is achieved through the use of the visual methods of community mapping and Photovoice.

## Aim of the study

The aim of the study was to explore how children construct and assign meaning to the “self” within two urban communities of the Western Cape through the use of methods that capture the children's perspectives on their well-being. The study further aimed to provide recommendations to inform further research and practice aimed at improving children's self-concept and its influence on children's well-being.

## Method

### Child participation

This study utilized a child participation framework in order to provide an in-depth exploration of the participants’ subjective experiences and understandings of the self. The child participation framework was chosen due to the limited research related to how children think and feel about aspects connected to their well-being. This is largely due to the current trend in studies of children in poverty that often look to the quantifiable aspects and depict children as victims, but often exclude their perspectives (Tekola, Griffin, & Camfield, 2009). Child participation research contributes towards a shift from an economic view of poverty to a multidimensional understanding, which promotes a holistic view of children's experiences of both well-being and adversity that could be used to inform more integrated interventions (Crivello, Camfield, & Woodhead, 2009). Child participation is further supported by current legislation on Children's rights as stated in articles 12, 13, and 14 of the United Nations Convention on the Rights of the Child, which emphasizes children's right to have a voice in the decisions concerning their lives, have their opinions taken into account, and be able to express their opinions in an age-appropriate manner (United Nations General Assembly, 1989). Research relating to well-being should place the child at the centre in order to understand the aspects that children value in their lives. Furthermore, a child-focused perspective creates a better understanding of children's experiences and perceptions along with opportunities for the children to be engaged in meaningful ways (Camfield et al., 2009).

### Research context

The research project took place in two urban communities located in the Cape Flats region of Cape Town, South Africa. The communities of Lavender Hill and Khayelitsha were selected in order to capture the diversity of the childhood experience within the Cape Flats due to their diverse spatial, cultural, and historical context. Both communities were formed as a result of Apartheid's system of “forced removals”; Lavender Hill was developed as a township designated to “Coloured” residents, while Khayelitsha was allocated to “Black” residents. The structural living conditions have an additional negative impact on the well-being of the residents and are typically characterized by poor infrastructure and service delivery, with high levels of crime and violence, substance use, and unemployment (Savahl et al., 2013). Lavender Hill is faced with the challenge of high density households, while only 45% of Khayelitsha households live in formal dwellings, the majority of the residents living in shacks in informal settlements (City of Cape Town, 2013b). Children in the communities are faced with the ongoing struggle of community violence, with the constant threat of victimization having a profound impact on their physical and psychological well-being. Despite the complexity of challenges faced by the residents of the communities, the strong culture and commitment of the residents within the communities have provided a support system for many of its children. There are a number of community-based and non-governmental organizations (NGOs) conducting programmes aimed at improving the lives and well-being of children in these communities.

### Participants and sampling

The study included a total of 54 participants between the ages of 9 and 12 years (26 females and 28 males). This age group was chosen due to the limited research available on the self-concept amongst children in the developmental stage of middle childhood (Benninger & Savahl, 2016) and marks an important period of life for the prevention of psychosocial challenges and for the promotion of mental health and wellness (Aber, Brown, & Jones, 2003).

Purposive sampling was utilized to select four groups of participants from two local primary schools and community organizations (two groups from each community). The criteria for participation were that the participants had to be: (1) between the ages of 9 and 12, (2) residing in one of the participating communities, and (3) presenting with a willingness and time to participate in the study.

#### Child reference group

Central to the study was the inclusion of child reference groups. Two groups were formed, one in Khayelitsha (three boys, two girls) and one in Lavender Hill (three girls, two boys) between the ages of 9 and 12 years with the brief of acting as co-researchers and community consultants throughout the entire qualitative research process, including the focus group discussions, Photovoice, and community mapping. Moore, Noble-Carr, and McArthur (2015) noted the importance of using children's reference groups as a strategy for participation and for co-reflexivity. They specifically point to the usefulness of child reference groups in facilitating the exploration of deeper levels of meaning and interpretation. It was therefore important to ensure the study was as inclusive as possible regarding the participation of the children and that their ideas and opinions were meaningfully incorporated throughout the research process. The child reference group participants were trained in qualitative research methodology and co-conducted the research amongst an additional 58 children within their respective communities. The children were selected from two community-based organizations in which the researcher was involved based on the credentials of being the appropriate age, being available for the weekly research sessions, and having an eagerness to participate. The child reference group participants assisted with every stage of the research process with the exception of the conceptualization of the research topic, which formed a part of a larger PhD dissertation.

### Data collection: Photovoice and community mapping

The research drew upon the visual methods of community mapping and Photovoice in order to provide a more holistic understanding of the research phenomenon. Visual methods are beneficial for participatory research with children because they build upon various forms of communication and provide the opportunity for children to choose what they want to contribute and express within the study (Darbyshire, MacDougall, & Schiller, 2005). A range of methods is additionally important for research with children because it takes into account their developmental stages (Ennew, 2009) and various levels of literacy (Crivello et al., 2009). Furthermore, using a range of research methods with children helps to triangulate the data and build a clearer picture of the phenomenon under investigation (Morrow, 2001). For example, Young and Barrett (2001) used multiple methods such as photos, drawings, mapping, and focus group discussion to capture the socio-spatial environment of street children in Kampala, Uganda. Another example is reflected in the study by Clark and Moss (2005) who used photography, interviewing, observations, and drawings amongst early childhood participants as a means of involving children in the redevelopment of a preschool. The current study incorporated a broad range of children's capacities for expressing themselves, specifically through the use of the data collection techniques of Photovoice, community mapping, and group discussions.

#### Photovoice

Photovoice is a qualitative research methodology that utilizes the process of producing photographic images to address a particular community concern. Photovoice is an effective technique to use with children because it provides the opportunity for the participants to gain power through incorporating their personal voice, experience, and language (Hergenrather, Rhodes, Cowan, Bardhoshi, & Pula, 2009). The photos are used to generate discussions around the research topic and within the final data analysis. In the current study, the participants were asked to take photos based upon the following brief: *What in your life influences how you think and feel about yourself? What influences how children in the community think and feel about themselves*? The participants were placed in pairs sharing a disposable camera with 27 photos, which they were allowed to take home, with the request to return the camera in 1 week.

Following the return of the cameras, the researcher had the photos developed and facilitated a Photovoice debriefing session. During this session, the photos were visually displayed and the group was asked to comment on the photos. Questions to stimulate discussions about the photos included: *What do you see here? What is happening in this photo? How does this relate to how you or other children in your community think and feel about yourself and themselves? What can we do about it?* The children then had the opportunity to present their photos or to select their favourite photos and explain why they chose the photo. This technique was useful because it allowed the children who did not have photos to be included in the conversation. The children who felt less comfortable speaking were provided with the option of writing descriptions of their favourite photos.

#### Community mapping

The community mapping activity was used to gain a deeper understanding of the regular social and environmental influences that could influence the self-concept of the local children. Mapping exercises entail the drawing and discussion of a map of the social and physical environment of children, which creates a valuable means for capturing children's perceptions while encouraging free responses and individual interpretations (Darbyshire et al., 2005). During the community mapping sessions, the participants were divided into groups of three or four and asked to draw a map of their community, specifically focusing on the places where they regularly engaged and the spaces that influenced how children thought and felt about themselves. The participants were then provided with the opportunity to explain the drawings through the use of writing or verbal response, which provided a rich description of the spatial and environmental influences on their self-concept. The maps were additionally used to stimulate further discussion around the changes that needed to be made in order for their community environment to promote a healthy self-concept. The community mapping exercise was useful for creating an alternative avenue for children to express themselves and for triangulating research data from the Photovoice sessions.

### Data analysis

The analysis proceeded by means of a thematic analysis as proposed by Braun and Clarke (2006) which included six phases: (1) familiarizing yourself with the data, (2) generating initial codes, (3) identifying themes, (4) reviewing themes, (5) defining and naming themes, and (6) producing the report. During the first phase, the primary author transcribed the data, read and reread the data, and noted initial ideas. Initial codes were systematically created from the interesting features of the data. Colour coding the codes within the text and creating a visual map of the codes assisted with this process. The codes and data relevant to the codes were then collated into potential themes utilizing a table format. The next phase included re-reading through the entire data set to ensure the themes accurately represented the data and to add additional data within the existing themes that may have been overlooked. After the themes were defined and further refined, a detailed analysis was written for each individual theme and included within the final report. The child reference group was consulted in order to check for the accuracy of the themes and the final analysis was verified by both the child reference group and the research participants in the form of a child-friendly report in order to ensure that their voices were accurately represented.

### Procedure and ethics

The research was approved by the Senate Research Committee at the university where the researchers are based. The researchers partnered with two local NGOs, Waves for Change and Philisa Abafazi Bethu, who assisted with the process of participant recruitment. An initial session was held with the selected participants, introducing the aims of the study, expectations of participation and the ethics principles of informed consent, voluntary participation, confidentiality, the freedom to withdraw without consequence, permission to audio record, and the academic use of the data. The participants and their guardians were provided with an information sheet explaining the details of the study as well as consent forms for both the guardian and the participant. Only those who provided both signed consent forms participated in the study. The discussions were conducted in the school hall at a local community centre after school hours by the primary researcher with the assistance of a local community youth care worker. The participants were provided with food and refreshments during the sessions, received age-appropriate research skills training, and a closing party. Counselling was available for any of the participants who experienced emotional discomfort. The recordings were transcribed and translated by an external transcriber, verified by a member of the supervisory team, and securely stored. Moore et al. (2015) highlights the ethical importance of involving the participants in the post data analysis phase of a research project (Moore et al., 2015). While the child reference group assisted with the data analysis stage of the research process, the study findings were further validated by the groups of participants who were provided with a child-friendly report.

The following guidelines were followed during the Photovoice sessions for the privacy and safety of the participants and the community members. Two written consent forms were administered to the participants. The first form followed the university institutional review boards’ ethics protocols adopted to Photovoice, which provided a detailed explanation of the participants’ voluntary involvement in the Photovoice process. The second consent form was for the approval of the publication of photographs or for the utilization of photographs in the final report and the dissemination of research findings to relevant stakeholders. The participants participated in a Photovoice training session, in which they were instructed to follow the ethical guidelines for Photovoice that included a discussion of the participants’ responsibilities to respect the privacy and rights of others, a dialogue that yielded specific suggestions and ways to respect others’ privacy and rights, and the emphasis that no picture is worth taking if it begets the photographer harm or ill will (Wang, Cash, & Powers, 2000, p. 87).

**Image 1:**
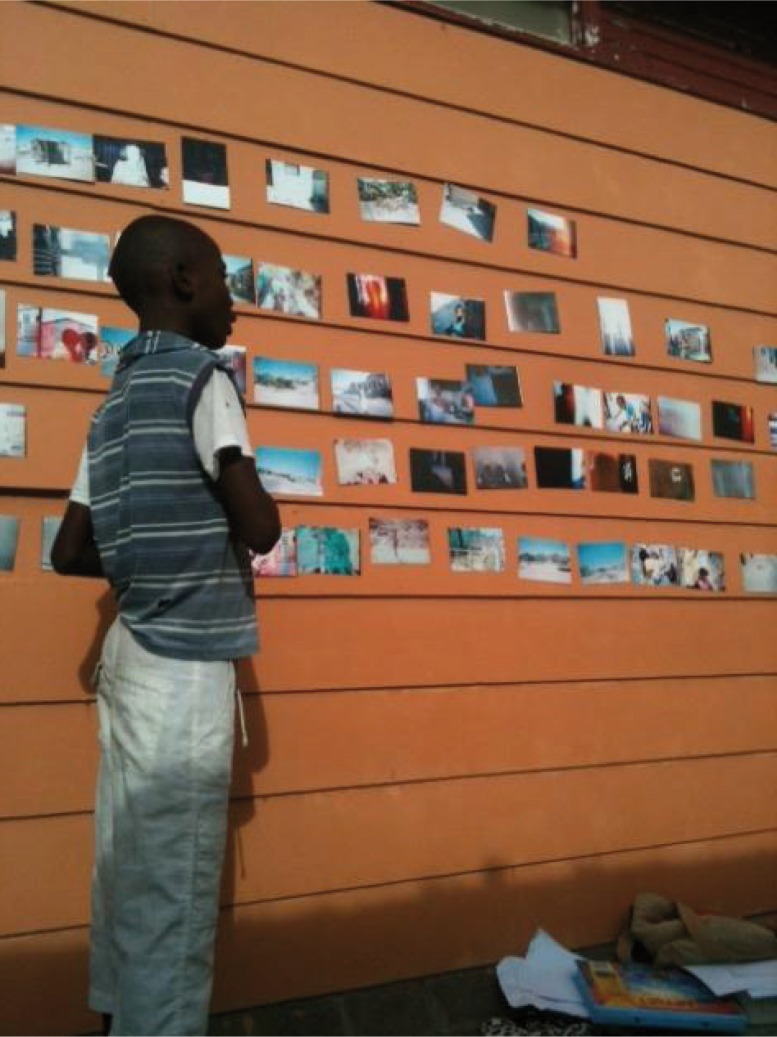
Participant discusses the significance of his photos during the Photovoice discussion session.

Maglajlic (2010, p. 213) describes the most important ethical issue in participatory research with children to be “What will happen with the children's efforts once adults gain relevant research knowledge? How will they be supported to continue their work?” The primary researcher was actively engaged with the local community organizations and schools, attended regular community forums, provided life skills and mental health education, resource development, and facilitated after-school programmes within the first community for 4 years and the second community for 1 year prior to the start of the research project. This allowed the researcher to provide timely feedback of the results to a variety of community stakeholders so that the findings could be utilized by the larger community to contribute to improvements in local child well-being. The findings were shared at parent-teacher meetings and provided a valuable contribution to the Waves for Change programme curriculum.

## Findings

The identified themes related to the participants’ self-constructions and meaning assignations included feelings around safety, children's spaces, and social connectedness.

**Image 2:**
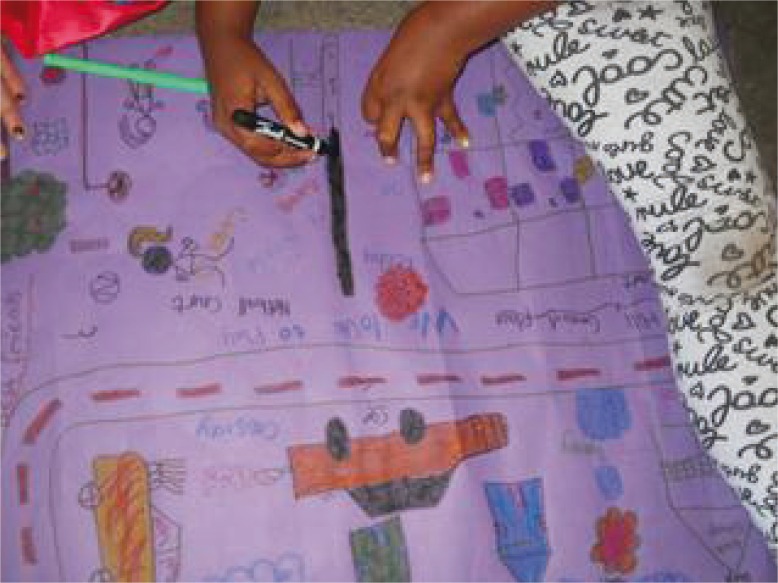
Community mapping activity.

### Feelings around safety

Central to the community maps, photographs, and the discussions around the self were the feelings that the participants associated with their personal safety and the safety of other children. The feelings associated with the visual data included anger, sadness, fear, strength, bravery, silliness, and calmness. The discussions around their feelings revealed an underlying sense of helplessness and vulnerability, which they associated to their identity as children. The streets within the participants’ neighbourhoods were characterized by violence with limited spaces of safety. These safe spaces could only be accessed through “dodging bullets, cars, and gangsters.” The maps and descriptions appeared to be a reflection of the participants’ internal world, where, in the absence of safety, they struggled to form a secure sense of self. In contrast to their chaotic reality, the children longed for a community where they could feel calm and safely play outside. Often, a sense of fear was depicted through the narratives of the participants’ first-hand accounts of violence or through the sharing of stories of the other children in the community:

Male Participant 1: There was a kidnapping, this has affected us a lot because the kid who was kidnapped had his body cut up, put in a black bag, and buried … This spreads a lot of fear amongst the kids.

Male Participant 2: There was also a poison and a lot of kids died because a man poisoned children in the community, this makes it scary to be a child.

(Khayelitsha, Group 1)

**Image 3:**
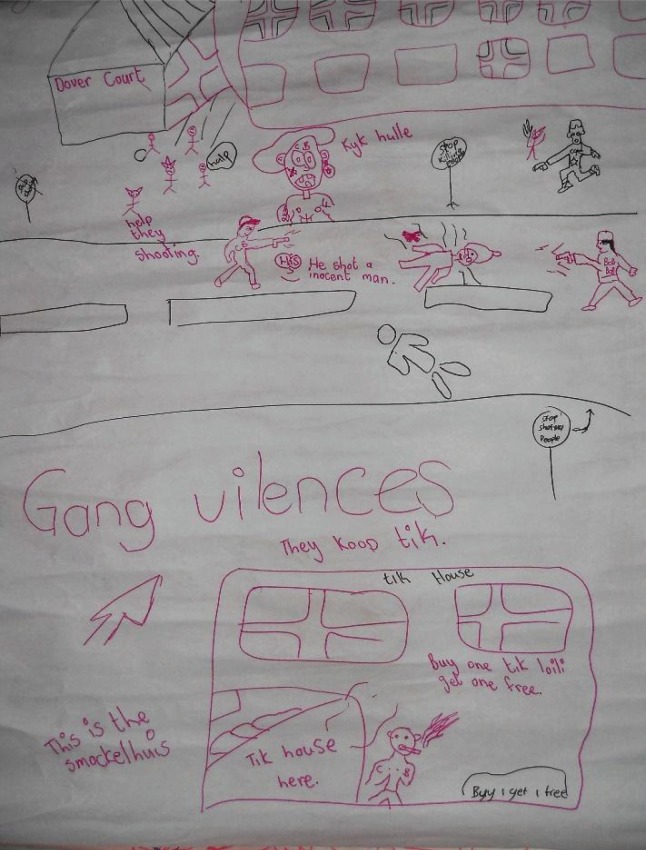
Community Map identifying the issues of violence within the community which negatively influence the self-concept of the local children (Lavender Hill, Group 1).

The participants’ discussions of their personal accounts of violence contributed towards a collective identity as children constructed around survival. A preoccupation with survival made it challenging for the participants to reflect on the deeper meanings associated with their selfhood. The violence which the participants reported included sexual abuse, bullying, abuse by adults in the home, or victimization by the gangsters. The participants had seen first-hand or heard of violent encounters on a regular basis and related and built off of each other's narratives as a means of making sense of their individual and collective identities. In addition to a “collective survival narrative,” an underlying sense of helplessness and vulnerability manifested itself within the discussions around the maps and the photographs:

Facilitator: so there is a lot of pictures of fighting, how does that make kids feel about themselves?

Male Participant # 1: They feel angry.

Facilitator: Why do they feel angry?

Male Participant #2: They feel sad.

Facilitator: Why do they feel angry, why do they feel sad?

Male Participant #2: Because they can't fight for themselves.

(Khayelitsha, Group 2)

**Image 4:**
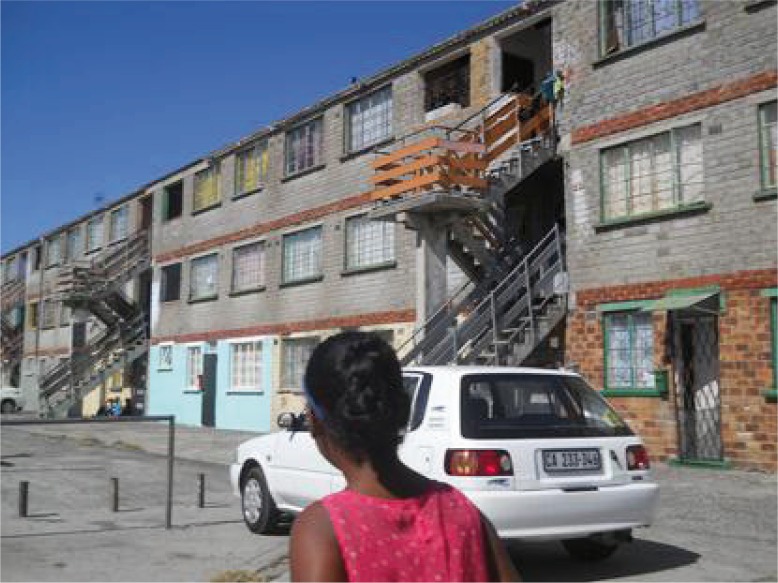
“This is the court where we live. Here is where we see the dead bodies. Sometimes they are left there for days” (Lavender Hill, Group 2).

In the above account, the participants discussed the vulnerability related to their social position in the community where they *can't fight for themselves*. They struggled to make sense of themselves as children because they felt the surrounding violence had deprived them of the central qualities that comprised their idealized child identity; the ability to be protected and play safely outside. This sense of vulnerability was often tied to their susceptibility to the gang violence in the community: “When the gangsters want to rob us we just give them everything we have” (Khayelitsha, Group 2).

**Image 5:**
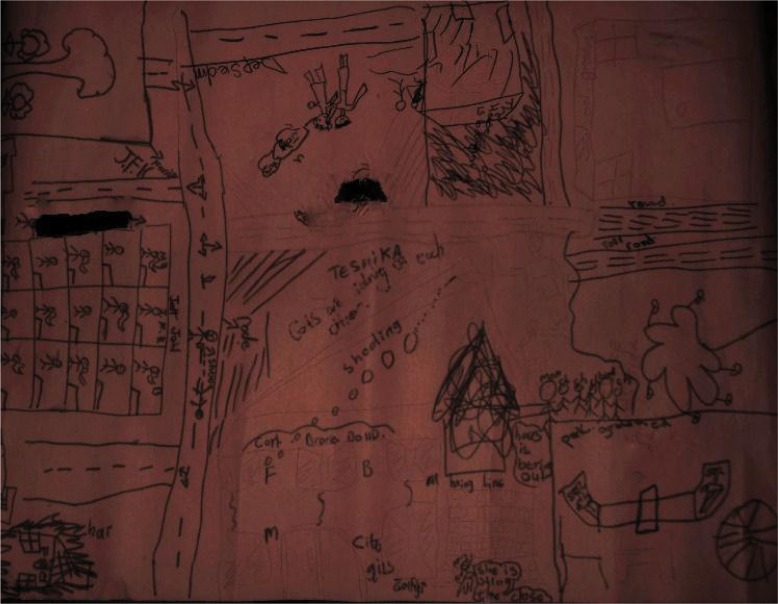
Lavender Hill Group 1, Community Map.

Connected to their feelings of helplessness was a sense of dependency on the adults for protection and support. “The children are feeling unsafe. If they want to play outside they need someone to protect them” (Lavender Hill, Group 1). This protection was not always easy to find, leaving the children with an uncertain sense of safety and a lack of trust in the adults to protect them. This led to an absence of a secure base to explore the self and contributed negatively towards the participants’ sense of self-worth: “Building more safe places for children will make children think positively about themselves because they will feel like adults are thinking about them and care about the things that are important to them” (Lavender Hill, Group 2). For some of the participants, feelings of hope were constructed around the belief that the adults would eventually come and help protect them:

Female Participant: In our place, they can't see what's happening to us … the gangsters are shooting by us … And when they look at our pictures they will see that this is true what the children are drawing.

Facilitator: So they will come and they will see that what you are drawing is true, that this is really happening.

Male participant: And then they will stand up!

Female participant: We are not just children, we are also people!

(Lavender Hill, Group 1, See Image 5)

In the above account, the participant attempted to defend her sense of self-worth within her experiences of violence. Even though she knew that she was perceived to be a child by others, she fought for her right to be viewed as an equal person whose needs were important. She further expressed her hope that the adults in positions of power would one day listen and change the conditions for the local children.

**Image 6:**
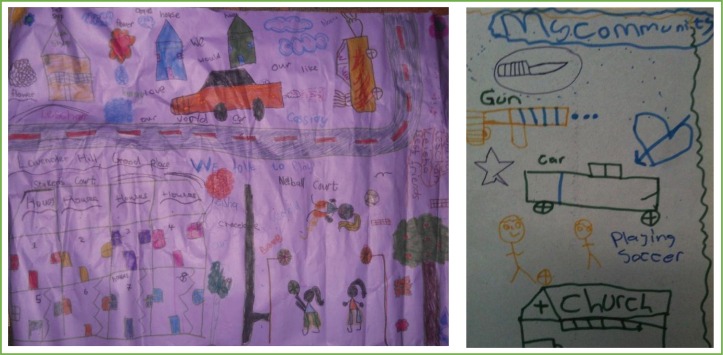
Maps created by the participants regarding the spaces which support and those which challenge the way children think and feel about themselves. (Khayelitsha group 1 (Left), Lavender Hill group 2 (right)).

Many of the participants chose to take photos of the aspect of their lives, which provided them with a sense of safety and calmness, as seen in the following quote: “What I like about this photo is that this puppy is safe. I like to be safe” (Khayelitsha, Group 1). Another participant similarly referred to young children as important contributors towards his sense of security: “It's good to have young children around because young children are safe, they are not using guns” (Khayelitsha, Group 1). When asked to describe the changes they would make to their community to assist the way children think and feel about themselves, the participants explained that they would like the community to be a safer and calmer place. This meant putting a stop to the shooting, drugs, and other forms of violence that took place on the street and at times in the home.

For some of the participants, their on-going exposure to aggressive behaviour contributed towards a self-concept formed around violence. This was often associated with feelings of anger, strength, and a need for survival:

Facilitator: Why do you think there is so much fighting? What makes you feel so angry?

Male participant: Because we are kids, we are angry because we are kids.

Facilitator: Why do you get angry?

Male respondent: Because I'm strong.

In the above account, the participant associated anger and the use of violence to be an innate and natural component of his self-concept as a child and a sign of his strength. This could be related to the status of the gangsters in his community, who gained their power and wealth through the use of violence. Violence served both as an ego defence mechanism as well as a strategic defence mechanism to protect the participants’ physical and psychological safety, while preserving their self-integrity. These feelings were also related to the participants’ strong sense of empathy which they felt for the other children in the community, especially those who were victimized by the violence: “sometimes when they fight with the guns, the bullets pass through and kill children, this breaks our hearts” (Khayelitsha, Female, Group 1). The unpredictability of the environment was described as leading to children being continuously on edge and compromising their sense of self.

**Image 7:**
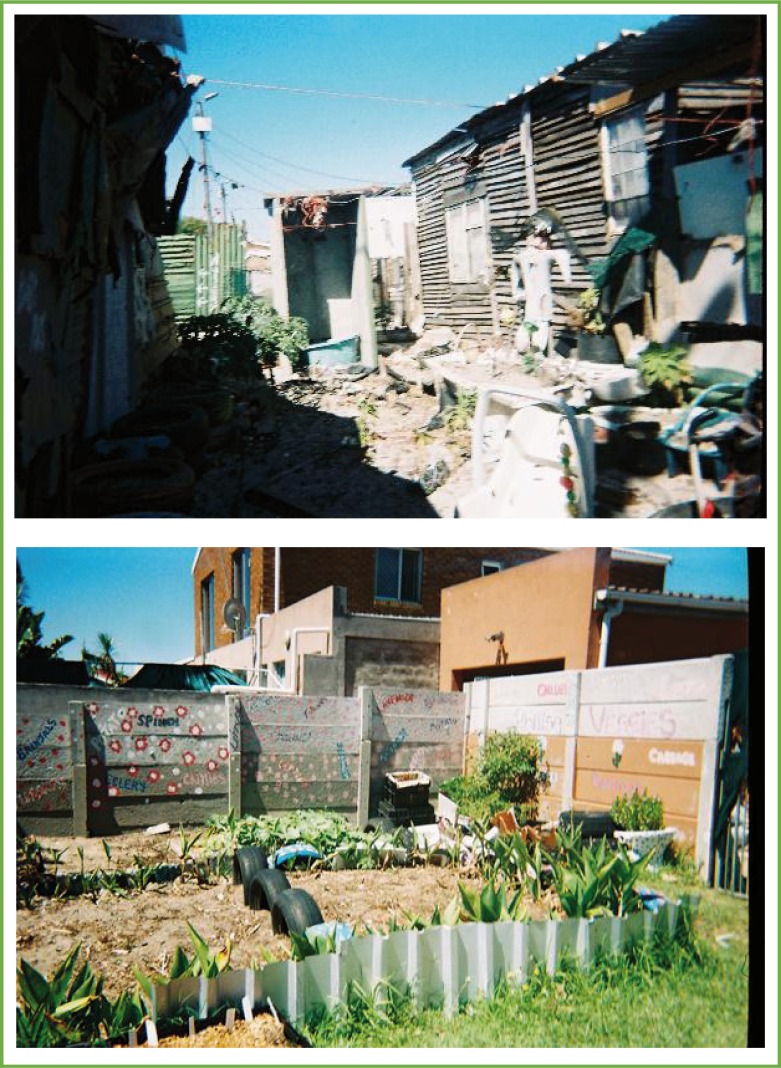
Photos taken to represent the contrasts within the physical environment which influence the way children think and feel about themselves.

### Children's spaces

The spaces within the community where children engaged were portrayed in two contrasting categories; the good versus the bad spaces. The good spaces included the schools, community centres, children's programmes, churches, houses of friends and family, and sports fields. The bad spaces were identified as the streets in their communities which they must pass through in order to reach the spaces of safety. Additional bad spaces identified were the known drug-dealers houses, dump yards, and at times their own homes. Shops were identified to be a neutral and necessary space within the community because it provided people with the goods that they needed without having to travel very far. Although the houses were identified to be safe spaces, especially when referring to the shelter from the bullets, they were also considered spaces of imprisonment and boredom, which deprived the local children of their right to play safely.

The housing infrastructure in the participants’ communities consisted of high density and overcrowded apartments or shacks, where there were limited spaces for play. The children evocatively referred to being “imprisoned” in their homes, which forced them to forfeit an important aspect of their child identity; the ability to play safely outside. The notion of play was brought up regularly by the participants as a central component of their child-identity. The denial of the opportunity to play compromised the way in which they made sense of themselves and their surroundings. This also left the children feeling distressed about themselves and their situation in the community. It was, however, apparent that, despite adverse circumstances, the participants were able to seek out and create their own opportunities for play. Although for some of the participants, the violent identity of the gangsters was reflected in their play; for others the ability to create peaceful opportunities for play allowed them to escape their violent surroundings and reflect on their self-potentialities.

**Image 8:**
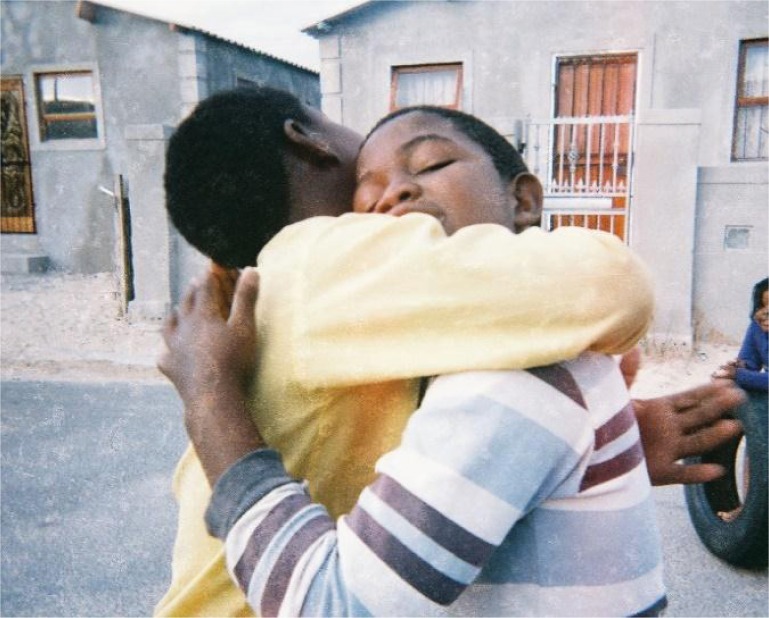
Photo taken to represent the positive influence which friends can have on the way children think and feel about themselves (Khayelitsha, Group 1).

Other participants mentioned the children in their community who had homes full of trash felt bad and ashamed about themselves and the area where they lived. In addition to the homes, dirty public spaces were considered dangerous for children and related to feelings of shame. In contrast to the dirty spaces, the participants described their appreciation for the green spaces within the community, such as the gardens, which allowed children to develop a sense of pride and self-efficacy.

The school was identified to be a space in the community that contributed positively to the self-concept of the local children. This positive contribution was attributed to the school's provision of safety, opportunities for learning, and for health: “My school is very nice, it's nice to be there because it's healthy, you do things you should do” (Khayelitsha, Group 1). The church was also described to be a good place for children because it assisted them in making good decisions. For many of the participants, the participation in church-related activities allowed them to create a sense of self connected to positive and pro-social behaviours.

### Social connectedness

Although the maps were largely constructed around images of violence, the majority of the photos depicted the positive aspects of the participants’ lives that were associated with their networks of social support. This included close family and friends, who were described to positively contribute towards the participants’ self-constructions. Having a friend to play with and one who you could talk to was especially important for making them feel happy and safe. A social connection through activities such as music and surfing with friends was identified as an important aspect of the participants’ self-identity and contributed towards feelings of happiness and pride. In addition to friends, the support of family was frequently mentioned as an important contributor towards a positive sense of self. An example is seen in a photograph taken of a participant's grandmother cooking supper. The participant explained, “I like this photo because it is nice when someone cooks for you” (Lavender Hill, Group 2).

**Image 9:**
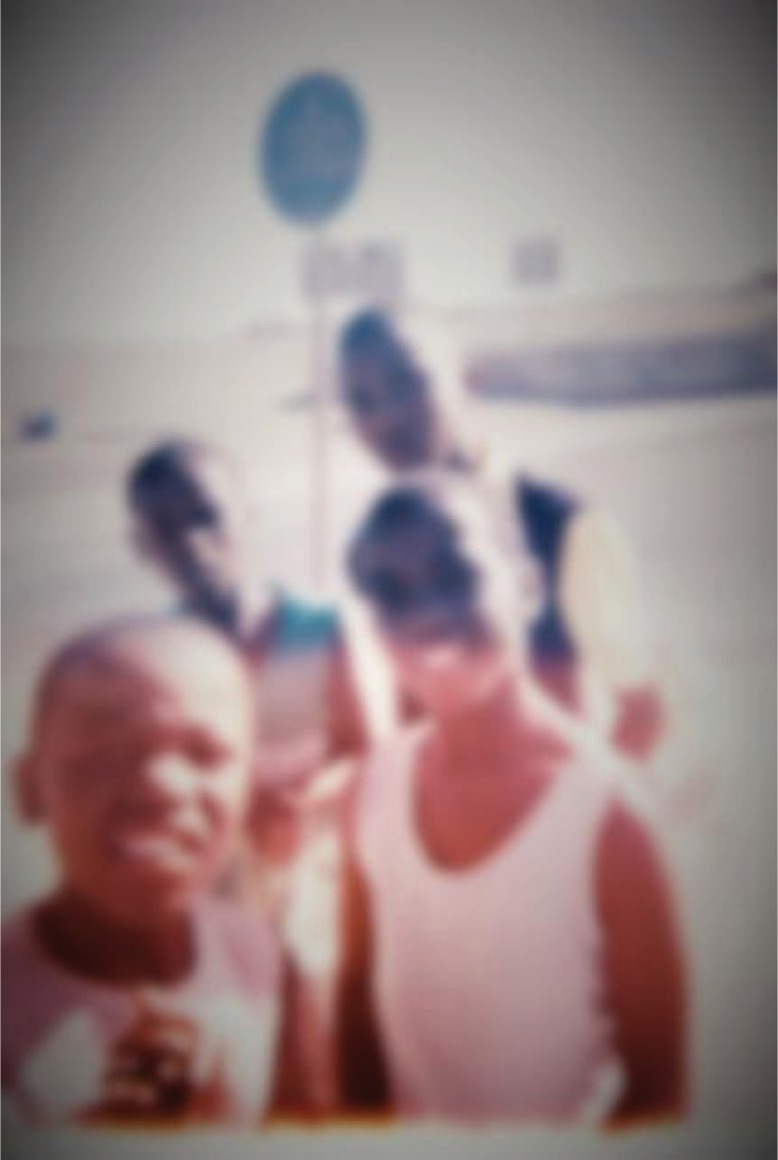
“Those are my friends, they also make me happy. If someone wants to hurt me, they always protect me” (Khayelitsha, Group 2).

Other photographs revealed the negative influences that peers and older youth could have on a child's self-concept and behaviour. One participant, for example, selected a photograph of two young children pretending to sniff glue and explained the concern he has for the young people in the community who engaged in such behaviours. As seen in the photo description in Figure 8, many of the participants were concerned about the impact which the older youth were having on the identity and behaviour of the younger children: “Those children are not able to think or feel anything because of the drugs. They can't even make goals for their future” (Khayelitsha, Group 1). Although the gangsters were identified as a threat to the participants’ sense of self and security, many of the participants associated the gangster identity to positive characteristics such as respect and praise.

## Discussion

Feelings of safety, social connectedness, and children's spaces all played a central role in the way in which the participants constructed and assigned meaning to the “self.” A sense of safety was not only expressed to be a “non-negotiable” for the participants’ well-being, but a “non-negotiable” for a stable self. This key finding resonates with those of a qualitative study conducted by Savahl et al. (2015) who found “personal safety” and a “stable self” as “non-negotiable” components of adolescents’ well-being. In the current study, the lack of safety resulted in feelings of chaos, anger, and sadness and compromised the participants’ sense of self-worth, especially as they compared themselves to their idealized child identity constructed around the ability to be cared for and to play safely. Although the majority of the participants portrayed violence in a negative manner, others justified its use as a means of gaining social and economic capital. This is in accordance with the research by Parkes (2007), which portrayed violence for young people in South Africa to carry multiple meanings which resulted in multiple consequences. In the current study, such consequences were apparent in the multiple self-constructions formed around the participants’ experiences and understandings of violence.

**Image 10:**
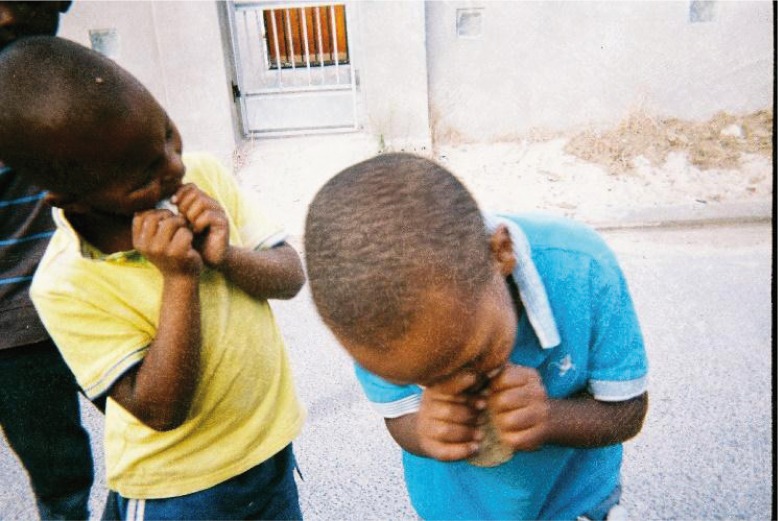
“My best picture. What I like about this picture, it's when little kids try something wrong. Smoking is not going to take you places. If you start smoking when you are still young your life is in danger. The older boys will teach you bad things. Do not try it because you will love it. Always stand up for yourself and your rights. Do not risk your life!” (Khayelitsha, Group 1).

In addition to the “non-negotiable” need for safety, social support played a crucial role in the development of a positive sense of self. Research has shown social support to contribute towards a healthy self-development in the face of adverse environmental circumstances (Timberlake, 1994). Support in the form of accepting relationships has been shown to contribute to self-growth and feelings of security and freedom (Cockle, 1994). The existence of role models with whom young people can identify could also contribute towards a sense of hope in relation to one's future self (Abrams & Aguilar, 2005). In this study, the availability of social support contributed towards a positive sense of self, although this did not necessarily result in positive behaviour. Engaging with friends allowed the participants to feel socially connected to others which resulted in feelings of happiness and acceptance. The activities which the participants engaged in with their friends were influential in their self-constructions, even if these were deemed to be anti-social. The availability of supportive adults and older youth contributed towards feelings of self-worth and a sense of self-efficacy. Abusive adults and older youth, on the other hand, contributed towards feelings of helplessness and vulnerability because the participants’ size and social status prevented them from being able to defend themselves or change their situation. This was reflected in both the maps and the photos of the participants, where there appeared to be a conflict between the people in close proximity who supported the self and those who compromised it.

In addition to their social connections, the children's self-identity was expressed to be closely linked to the spaces where they regularly engaged. Ellis (2005) described place to be significantly seen as a means through which people construct an identity. Proshansky, Fabian, and Kaminhoff (1983) further support this notion within the theory of place-identity, defined as the subjective sense of self which is conceptualized not only through one's social relationships but through one's relationships to the various physical spaces that define a person's day-to-day life. Self-identity growth is therefore related to the development of a meaningful place-identity. This was evident in the participants’ photos, maps, and discussions, where they commonly spoke about themselves in relation to the meaningful spaces where they preferred to spend their time. Safe, green, and clean spaces in the community were valued by the participants and made them feel good about themselves and connected to their area, while dirty spaces had the opposite effect. Spaces where the participants could engage in activities such as gardening allowed them to develop a sense of self-efficacy and pride in their ability to improve their community. The spaces which provided activities for learning new skills were actively sought out by the participants because these spaces instilled a sense of confidence in themselves and their abilities, and provided them with the opportunity to develop a sense of self. The importance of the connection between the sense of self and place is similarly reported by (Adams & Savahl, 2016), who found that children's self-concept is influenced by their engagement in safe natural spaces.

Although space and place contributed towards the development of the self, the participants viewed their day-to-day spaces to be controlled by the adults. Ataöv and Haider (2006) found similar responses amongst street children in Turkey. They concluded that the public space often marginalized children and that their inclusion was imperative in the research, planning, and management of the public space. Their research highlighted the propensity to play, which children had in any space sufficient enough to meet their needs, although this was often obstructed by the adults. The participants in the current study expressed their desire for adults to take children seriously and to recognize their contribution towards society. This recognition would allow the participants to develop a sense of self as valued and capable members of the community. In accordance to the international and national legislation on children's rights, this meant including children in decisions related to their lives and providing them with the activities which the children felt were important. Amongst their responses was a need to prioritize the construction of safe spaces within the public sphere for children to play. The ability to play was not only an important contributor to the way the children felt about themselves, but also provided them with the opportunity to make sense of themselves, their abilities, and potentialities.

The visual methods of Photovoice and community maps provided a useful and engaging means of gathering data regarding the thoughts and feelings, which the child participants had of their social and physical environment and how these influenced their self-concept. The photos taken by the participants allowed for a collaborative and creative discussion around their self-constructions and provided a more holistic understanding of the various components that influence children's self-concept. The community mapping exercise was a valuable means of triangulating the data from the Photovoice activity, while providing the opportunity for the child participants to utilize drawing as a means of communicating their thoughts and ideas related to their self-concept. Although the final data analysis was verified by the child reference group and the participants based on the group discussions around the visual data, the analysis itself was conducted by the adult researchers, whose interpretations could be different than the interpretations of the participants. The adult-child power dynamics between the adult researchers and the child participants also posed a challenge to ensuring that the data collection and analysis accurately reflected the children's voices.

## Conclusions and recommendations

This study highlights the impact which the social and physical environment has on the way in which children construct and assign meaning to the “self” and how this influences their perceptions of their well-being. The availability of safety, social support, and nurturing children's spaces all contributed towards the ideal social environment for fostering a child's sense of self. Central to the development of a healthy and stable self-concept was the requirement for a sense of safety and security in the broader social environment. Experiences of violence and abuse posed a threat to the participants’ sense of self, while experiences of love and support from others within a safe space helped buffer the negative impact of violence on the self. The meanings assigned to the participants’ day-to-day spaces contributed towards their sense of self, especially in the form of their sense of place-identity. This provides implications for intervention programmes aimed at improving children's well-being to take into consideration activities aimed at improving children's self-concept. This includes the construction of safe spaces for children to play, learn, and form meaningful relationships. Interventions must also look at the larger structural, community, and cultural factors that marginalize children and leave them with a sense of vulnerability and helplessness. One way to address this is through the recognition of children as citizens whose feelings, ideas, and experiences need to be taken seriously. Furthermore, interventions must focus on creating a culture in which children are valued and included in community building processes. The structural inequalities of the context in which the research took place cannot be overlooked, as these contribute towards the continuity of violence and poverty within the communities and the barriers towards the creation of safer and more secure environments for the local residents. Due to the contextual nature of the self, further research is necessary to understand the unique ways in which children construct and assign meaning to the “self” within diverse communities and spaces. This should include engaging and using age-appropriate tools such as Photovoice and community mapping, which allow for children to participate in a meaningful way and take into account children's various means of communication. Additional research is also necessary for the development and the evaluation of programmes aimed at improving the self-concept of children.
